# Diagnostic Performance of Clinical and Routine Laboratory Data in Acute Mesenteric Arterial Occlusion—An International Multicenter Study

**DOI:** 10.3390/diagnostics14232705

**Published:** 2024-11-30

**Authors:** Yasmin Soltanzadeh-Naderi, Annika Reintam Blaser, Martin Björck, Alexandre Nuzzo, Joel Starkopf, Alastair Forbes, Marko Murruste, Kadri Tamme, Peep Talving, Anna-Liisa Voomets, Merli Koitmäe, Miklosh Bala, Zsolt Bodnar, Dumitru Casian, Zaza Demetrashvili, Mario D’Oria, Virginia Dúran Muñoz-Cruzado, Hanne Fuglseth, Moran Hellerman Itzhaki, Benjamin Hess, Karri Kase, Kristoffer Lein, Matthias Lindner, Cecilia I. Loudet, Damian J. Mole, Sten Saar, Maximilian Scheiterle, Kenneth Voon, Jonas Tverring, Stefan Acosta

**Affiliations:** 1 Department of Clinical Sciences, Malmö, Lund University, 214 28 Malmö, Sweden; yasmin.soltanzadeh-naderi@med.lu.se; 2 Institute of Clinical Medicine, University of Tartu, 50090 Tartu, Estonia; annika.reintam.blaser@ut.ee (A.R.B.); martin.bjorck@uu.se (M.B.); joel.starkopf@kliinikum.ee (J.S.); alastair.forbes@ut.ee (A.F.); marko.murruste@kliinikum.ee (M.M.); kadri.tamme@kliinikum.ee (K.T.); peep.talving@ut.ee (P.T.); karri.kase@kliinikum.ee (K.K.); 3 Department of Intensive Care Medicine, Lucerne Cantonal Hospital, 6000 Lucerne, Switzerland; benjamin.hess@luks.ch; 4 Department of Surgical Sciences, Vascular Surgery, Uppsala University, 753 10 Uppsala, Sweden; 5 Department of Gastroenterology, IBD and Intestinal Failure, Intestinal Stroke Center, AP-HP. Nord, Beaujon Hospital, Paris Cité University, 75006 Paris, France; alexandre.nuzzo@aphp.fr; 6 Tartu University Hospital, 50406 Tartu, Estonia; annaliisavoomets@gmail.com; 7 Division of Acute Care Surgery, North Estonia Medical Centre, 13419 Tallinn, Estonia; 8 Estonian Genome Center, Institute of Genomics, University of Tartu, 50090 Tartu, Estonia; merli.koitmae@ut.ee; 9 Institute of Mathematics and Statistics, University of Tartu, 50090 Tartu, Estonia; 10 Hadassah Medical Center, Faculty of Medicine, Hebrew University of Jerusalem, Jerusalem 9190500, Israel; rbalam@hadassah.org.il; 11 Letterkenny University Hospital, Letterkenny F92 AE81, Ireland; zsolt.bodnar@hse.ie; 12 Department of General Surgery, Nicolae Testemitanu State University of Medicine and Pharmacy of the Republic of Moldova, MD-2004 Chisinau, Moldova; dumitru.casian@usmf.md; 13 N. Kipshidze Central University Hospital, 6160 Tbilisi, Georgia; zdemetr@yahoo.com; 14 Division of Vascular and Endovascular Surgery, Department of Clinical Surgical and Health Sciences, University of Trieste, 34127 Trieste, Italy; mario.doria88@outlook.com; 15 Virgen del Rocío University Hospital, 41013 Sevilla, Spain; virginia.dm.87@gmail.com; 16 Stavanger University Hospital, 4011 Stavanger, Norway; hanne.fuglseth@sus.no; 17 Rabin Medical Center, University of Tel Aviv, Petah Tikva 4941492, Israel; moranhe@clalit.org.il; 18 University Hospital North Norway, 9019 Tromsø, Norway; kristoffer.lein@unn.no; 19 Universitätsklinikum Schleswig-Holstein, Campus Kiel, 24105 Kiel, Germany; matthias.lindner@uksh.de; 20 Hospital General San Martin de La Plata, Buenos Aires B1900, Argentina; cloudet@med.unlp.edu.ar; 21 Royal Infirmary of Edinburgh, Edinburgh EH16 4SA, UK; damian.mole@ed.ac.uk; 22 North Estonia Medical Centre, 13419 Tallinn, Estonia; sten.saar@regionaalhaigla.ee; 23 Azienda Ospedaliera Universitaria Careggi, 50134 Firenze, Italy; max.scheiterle@gmail.com; 24 Sarawak General Hospital, Kuching 93586, Malaysia; kenneth.voon@ummc.edu.my; 25 Department of Clinical Sciences, Lund, Lund University, 221 00 Lund, Sweden; jonas.tverring@med.lu.se

**Keywords:** acute mesenteric ischemia, arterial occlusive AMI, acute superior mesenteric artery, clinical diagnosis, laboratory markers

## Abstract

Background: There are no clinical or laboratory markers that can diagnose acute mesenteric ischemia (AMI) accurately. This study aimed to find differences in clinical and laboratory markers between arterial occlusive AMI and other acute abdominal diseases where AMI was initially suspected. Methods: This was a post hoc study of an international prospective multicenter study where data on patients with suspected AMI were collected. Independent factors associated with arterial occlusive AMI were evaluated in a multivariable logistic regression analysis. Results: The number of patients with arterial occlusive AMI was 231, consisting of thrombotic (*n* = 104), embolic (*n* = 61), and indeterminate (*n* = 66) occlusions. The non-AMI group included 287 patients, of whom 128 had strangulated bowel obstruction. Current smoking (odds ratio [OR] 2.56, 95% confidence interval [CI] 1.31–5.03), hypertension (OR 2.08, 95% CI 1.09–3.97), bowel emptying (OR 3.25, 95% CI 1.59–6.63), and leukocytosis (OR 1.54, 95% CI 1.14–2.08) at admission were independently associated with arterial occlusive AMI compared to the non-AMI group. Conclusions: This study found clinical and laboratory data to be associated with arterial occlusive AMI in patients with suspicion of AMI, which can possibly be of value in screening for arterial occlusive AMI at the emergency department. Further studies are needed to find more accurate diagnostic markers.

## 1. Introduction

Acute mesenteric ischemia (AMI) is a life-threatening condition with a high mortality rate. AMI, if not treated adequately, leads to intestinal gangrene and death [1]. One of the main causes of AMI is acute occlusion of the superior mesenteric artery (SMA): the artery which primarily supplies the small intestines and the proximal parts of the colon [1,2]. Arterial occlusive AMI has been shown to be the most common reason for AMI [1]. Occlusion due to embolism or thrombosis may have various onsets and affect different patient categories. Thrombosis—which often is secondary to extensive atherosclerosis—often has a more gradual onset with diffuse symptoms, whereas embolic occlusion—which oftentimes affects patients with atrial fibrillation—may have a more acute onset [1]. The classic clinical triad for acute SMA embolus includes abdominal pain with minimal clinical findings, bowel emptying with vomiting and/or diarrhea, and source of emboli. However, studies have shown inconsistencies in the frequency of these findings [1].

I-FABP, D-lactate, and citrulline were believed to possibly have diagnostic value in patients with AMI; however, a recent systematic review [3] found that those markers failed to differentiate patients with AMI from patients with acute abdomen from other causes. Importantly, one of the most common differential diagnoses of AMI is strangulating bowel obstruction (SBO), and a plasma biomarker differentiating these conditions would be of great value in the clinical workup [4]. Currently, computed tomography (CT) angiography is the gold standard of diagnosis in patients with suspected arterial occlusive AMI [1,5], but it is not without flaws [6].

The AMESI (Acute MESenteric Ischemia) study was a prospective international multicenter study which included patients with AMI along with suspected but confirmed non-AMI patients [7]. The study included patients from 32 hospitals globally.

The aim of this post hoc AMESI study was to find a possible combination of early clinical data and laboratory markers in patients with a suspicion of arterial occlusive AMI to facilitate patient selection for immediate CT angiography.

## 2. Materials and Methods

### 2.1. Study Design and Population

The prospectively collected study data were retrieved from the AMESI study [7]. The data included all patients with arterial occlusive AMI and patients with suspected but confirmed to be non-AMI. The study included patients over 18 years of age with suspected or confirmed AMI who were either admitted or transferred to 32 hospitals between 6 June 2022 and 5 April 2023. Patients with non-occlusive mesenteric ischemia (NOMI) and mesenteric vein thrombosis were excluded from the present study.

### 2.2. Definitions

Suspicion of AMI was raised by the local investigators in a patient with acute abdominal pain in the absence of another obvious diagnosis or critically ill patients in the intensive care unit with increasing plasma lactate levels and suspicion of non-occlusive mesenteric ischemia. AMI was confirmed by CT, endoscopy, surgery, histology, or autopsy in all cases. Patients with confirmed non-AMI (including SBO) comprised a collection of baseline data and hospital survival. Disability was categorized as the need for assistance in daily living activities (no/yes). Atherosclerotic disease was defined as previous ischemic heart disease, stroke, and/or peripheral arterial disease (carotid artery disease, lower extremity arterial disease). The Charlson comorbidity index [8] was calculated (mdcalc.com/calc/3917/charlson-comorbidity-index-cci). Bowel emptying was defined as diarrhea and/or vomiting.

### 2.3. Statistics

Categorical data are presented as numbers and proportions (%) and continuous data as medians with interquartile ranges (IQR). Variables associated with thrombotic or embolic arterial occlusive AMI, compared to non-AMI patients or SBO, in univariable analysis (*p* < 0.1), were candidates for inclusion in the multivariable logistic regression analysis, expressed in odds ratios (OR) with 95% confidence intervals (CI). The normality of data was assessed by the Kolmogorov–Smirnov test, and all tested continuous variables were log10 transformed due to skewed distribution and converted to Z scores before entering as covariates into a multivariable logistic regression model. Continuous data after multivariable testing were expressed per one standard deviation (SD) increment. A maximum of one covariate per ten arterial occlusive AMI events was allowed in the multivariable model [9]. No imputation of missing data was conducted, except when developing the prediction model (Supplementary Materials). The level of statistical significance was *p* < 0.05. IBM SPSS Statistics, version 28 (SPSS, Chicago, IL, USA) was used for statistical analysis. A clinical prediction model was developed using logistic regression modeling (Supplementary Materials).

## 3. Results

### 3.1. Comparison of Clinical Background Between Arterial Occlusive AMI and Non-AMI Groups

The total number of patients with confirmed acute occlusive arterial AMI was 231, of which all but 9 had SMA occlusions; 5 had occlusions of the inferior mesenteric artery and 4 of the celiac trunk. The total number of patients with suspected and confirmed non-AMI was 287, of which 128 had SBO. The etiologies of arterial occlusive AMI were thrombotic (*n* = 104), embolism (*n* = 61), and indeterminate (*n* = 66).

Compared to the non-AMI group, patients with arterial occlusive AMI were more often current smokers (*p* < 0.001), had more atrial fibrillation (*p* < 0.001), atherosclerotic disease (*p* = 0.020), hypertension (*p* < 0.001), myocardial infarction (*p* = 0.004), previous thromboembolic arterial events (*p* = 0.003), higher Charlson comorbidity index (*p* = 0.029), and more frequently used anticoagulant drugs (*p* = 0.004) (Table 1).

### 3.2. Comparison of Clinical Presentation Between Arterial Occlusive AMI and Non-AMI Groups

Patients with arterial occlusive AMI presented more often with diarrhea (*p* = 0.001), vomiting (*p* = 0.009), bowel emptying (*p* < 0.001), and shock (*p* = 0.031) than the non-AMI group (Table 2).

### 3.3. Comparison of Laboratory Data Between Arterial Occlusive AMI and Non-AMI Groups

**Figure A1:**
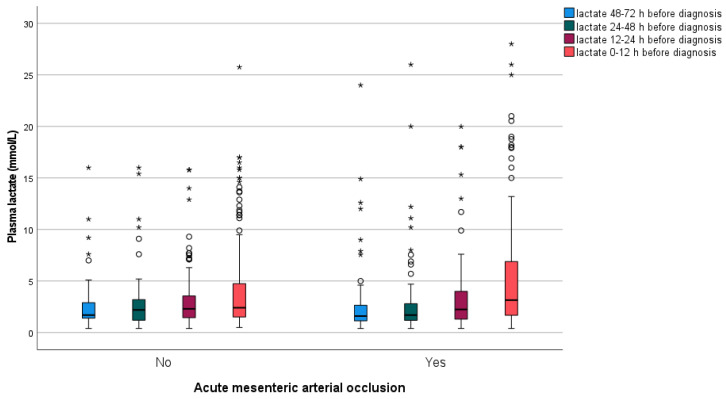
Box plot graph showing median and interquartile range (IQR) values of plasma lactate at different time ranges before diagnosis of arterial occlusive acute mesenteric ischemia (AMI) and non-AMI, respectively. The line across the box indicates the median, the box represents the interquartile range, and the whiskers are lines that extend from the box edges to the highest and lowest values, excluding outliers. Values more than 1.5 IQR’s but less than 3 IQR’s (o), and more than 3 IQR’s (*), from the box edges are labeled as outliers and extremes, respectively.

White blood cell count (WBC) (*p* < 0.001), C-reactive protein (CRP) (*p* < 0.001), and aspartate aminotransferase (ASAT) (*p* = 0.001) were more elevated in the arterial occlusive AMI group (Table 3). Base excess (*p* = 0.017) was lower in patients with arterial occlusive AMI. Plasma lactate levels at different time points prior to diagnosis were similar between the two groups when measured >12 h before verification of diagnosis but differed when measured 0–12 h before diagnosis, where plasma lactate levels were more elevated (*p* = 0.034) in the arterial occlusive AMI group (Appendix A, Figure A1).

### 3.4. Factors Associated with Arterial Occlusive AMI

Current smoking (OR 2.56, 95% CI 1.31–5.03), hypertension (OR 2.08, 95% CI 1.09–3.97), elevated WBC (OR 1.54, 95% CI 1.14–2.08), and bowel emptying (OR 3.25, 95% CI 1.59–6.63) were independently more associated with arterial occlusive AMI compared to the non-AMI group (Table 4).

### 3.5. Comparison of Clinical and Laboratory Data Between Arterial Occlusive AMI and SBO Groups

Univariable analyses were made comparing clinical and laboratory data in arterial occlusive AMI and patients with SBO (Appendix A: Table A1, Table A2 and Table A3). When entering hypertension, atherosclerotic disease, atrial fibrillation, current smoking, bowel emptying, WBC, CRP, ASAT, and D-dimer in a multivariable logistic regression model, only elevated D-dimer (OR 9.32 per one standard deviation increment, 95% CI 1.31–66.39; *p* = 0.026) remained as an independent factor associated with arterial occlusive AMI.

### 3.6. Comparison of Clinical and Laboratory Data Between Embolic Arterial Occlusive AMI and Non-AMI Groups

Univariable analyses were made comparing clinical and laboratory data in embolic arterial occlusive AMI (*n* = 61) and patients with the non-AMI group (Appendix A: Table A4, Table A5 and Table A6). When entering age, hypertension, atrial fibrillation, bowel emptying, WBC, and CRP in a multivariable logistic regression model, atrial fibrillation (OR 4.6, 95% CI 2.1–10.0; *p* < 0.001), bowel emptying (OR 3.1, 95% CI 1.4–6.9; *p* = 0.007), and elevated WBC (OR 2.1 per one standard deviation increment, 95% CI 1.3–3.4; *p* = 0.002) remained as independent factors associated with embolic arterial occlusive AMI.

### 3.7. Predictive Nomogram for Arterial Occlusive AMI Using a Combination of Clinical and Laboratory Data

A nomogram based on the variables hypertension, bowel emptying, current smoker, CRP, and WBC was constructed to estimate the probability of arterial occlusive AMI (Supplementary Materials). The model had moderate discriminatory performance on internal validation (C-statistic 0.718).

## 4. Discussion

In this prospective multicenter study, we found that smoking, hypertension, increased WBC, and bowel emptying (diarrhea and/or vomiting) were independently associated with arterial occlusive AMI when compared with the non-AMI group. Smoking was an unsurprising association since it is a known risk factor for atherosclerotic diseases [10], and the best medical treatment for arterial occlusive AMI includes smoking cessation [1]. Previous studies have shown a high prevalence of cardiovascular diseases, such as hypertension, in patients with arterial occlusive AMI [11]. Significant leukocytosis, a marker of severe abdominal pain [12], has been shown to be a predictive marker for transmural bowel necrosis [13,14]. Bowel emptying, which includes both vomiting and/or diarrhea, is a common symptom of AMI [15]. The pathophysiology of this is unclear but could possibly be explained by damage to the mucosal villi and bowel wall distention triggering bowel emptying [16]. The present study showed that bowel emptying was indeed more common than in the non-AMI group, even though a large share consisted of SBO. These associations could possibly be used in a prediction model, and an attempt was made to establish such a model to use when there is a clinical suspicion of arterial occlusive AMI. However, the model was not efficient enough and cannot be used in a clinical setting. Probable reasons for this are, among others, that the markers used in the model are not disease-specific enough and thus are present in several other acute conditions. A new attempt is warranted, preferably when more specific biomarkers are available.

The present study found no difference in the proportion of acute abdominal pain between the groups, whereas another recent report on patients with acute abdomen found that patients with arterial AMI had a higher proportion of sudden onset of pain and morphine-requiring abdominal pain [17].

New biomarkers could be helpful in diagnosing AMI earlier and hopefully increase survival rates. CT angiography is currently the favored method of diagnosis [1]; however, it is a diagnostic modality which has varied availability globally [18,19,20] and may pose a great cost for the patient and the healthcare system [21]. In some cases, it may also be overused [22]. A recent systematic review found that radiological predictors of transmural bowel necrosis may differ according to the various causes of AMI [23]. There are two prospective multicenter studies on biomarkers in the prediction of AMI in pipeline [24,25], where sequential blood samples will be collected in patients with suspected AMI to possibly find biomarkers that can distinguish between different AMI subtypes and their severity. In addition, there is another prospective cohort study searching for biomarkers of acute intestinal ischemia (https://www.ichgcp.net/clinical-trials-registry/NCT03518099, accessed on 18 September 2024). These studies will hopefully find new diagnostic biomarkers to be used as a complement or alternative to CT.

D-dimer was independently associated with arterial occlusive AMI compared to SBO, but it was not significantly associated with the whole non-AMI group. This finding could suggest that fibrinolytic activity [26] is lower in the SBO group.

Atrial fibrillation was independently associated with embolic AMI, which can be expected since atrial fibrillation is a known independent risk factor for other conditions caused by arterial embolisms [27]. It can be argued that in a patient with acute abdomen and atrial fibrillation, arterial occlusive AMI should be one of the differential diagnoses. The present study could indicate that increasing levels of lactate are a finding associated with the severity of the condition and a late-stage marker [28]. Plasma lactate may therefore not be used as an early diagnostic marker in the emergency department since normal lactate in AMI is a diagnostic pitfall [29]. On the other hand, persistently high levels of plasma lactate or increasing plasma lactate levels >200% after cardiovascular surgery should raise suspicion of the development of occlusive or non-occlusive AMI [30]. The subtype of AMI in this study by Mothes et al. was not declared, but it is well-known that most patients with AMI after cardiovascular surgery have non-occlusive mesenteric ischemia [1,30,31].

One of the main limitations is the post hoc analyses of the prospective data since some clinical variables, such as inability to pass gas, constipation/obstipation, and bowel distention, were not pre-specified and therefore missing, making a comparative analysis between groups impossible. Moreover, all the different sites chose their own blood samples in this pragmatic observational study without additional study-specific blood samples, which resulted in missing data for some laboratory data. Another limitation is that the study was composed of almost 60% of patients with AMI [7], indicating recruitment bias. The high proportion of patients with AMI among all suspected cases is highly unlikely since it is known that AMI is a complex and hard diagnosis to make on clinical grounds [1]. It is not exactly known how the different sites defined suspicion of AMI since it was not pre-specified in detail (to avoid interference with local clinical practices), making the inclusions more arbitrary and thereby leading to information bias. Also, not knowing all the diagnoses in the non-AMI group makes the data less applicable since there is a risk of confounding. Furthermore, not all blood samples were analyzed with the same methods: D-dimer, for example, had different assays provided by different manufacturers, which may affect the results [32,33,34]. Another limitation is that the timing of the blood samples in relation to symptom onset was unknown.

## 5. Conclusions

This study found pre-existing conditions and clinical and laboratory markers independently associated with arterial occlusive AMI compared to non-AMI. It was not possible to attain a well-performing prediction model, possibly due to the high similarity in both groups and markers that are not specific enough for the pathophysiology of the disease. However, this post hoc study is the first to be performed on arterial occlusive AMI on this scale and a well-needed step in the right direction to understand the disease better. Further prospective studies with predefined tests of novel plasma biomarkers in a core lab and biobanking of blood samples are needed to find more accurate diagnostic markers.

## Figures and Tables

**Table 1:** Comparison of clinical background between patients with arterial occlusive AMI and non-AMI groups.

	Arterial Occlusive AMI (*n* = 231)	Non-AMI (*n* = 287)	*p*-Value
Age, years (median, IQR)	71 (60–80)	69 (56–59)	0.14
Female gender (%)	100 (43.3)	138/284 (48.6)	0.23
Body Mass Index, kg/m^2^ (median, IQR)	24.4 (21.3–27.7) (*n* = 180)	24.7 (22.0–27.7) (*n* = 216)	0.56
Disability (%)	49/219 (22.4)	75/269 (27.9)	0.11
Current smoking (%)	70/180 (38.9)	51/225 (22.7)	<0.001
Atrial fibrillation (%)	84 (36.4)	65 (22.6)	<0.001
Atherosclerotic disease (%)	100/217 (46.1)	96/269 (35.7)	0.020
Hypertension (%)	166/224 (74.1)	169/280 (60.4)	0.001
Myocardial infarction (%)	48/221 (21.7)	33/274 (12.0)	0.004
Previous thromboembolic arterial event (%)	25/202 (12.4)	12/272 (4.4)	0.003
Charlson comorbidity index (median, IQR)	4 (3–6) (*n* = 212)	4 (2–5) (*n* = 268)	0.029
Anticoagulant drugs (%)	70/217 (32.3)	56/270 (20.7)	0.004
Antiplatelet drugs (%)	77/212 (36.3)	76/271 (28.0)	0.052
Statins (%)	76/213 (35.7)	85/270 (31.5)	0.33

AMI; acute mesenteric ischemia, IQR; interquartile range.

**Table 2:** Comparison of clinical presentation at admission between patients with arterial occlusive AMI and non-AMI groups.

	Arterial Occlusive AMI (*n* = 231)	Non-AMI (*n* = 287)	*p*-Value
Pre-hospital symptom duration, hours (median; IQR)	24 (9–48) (*n* = 172)	24 (6–48) (*n* = 190)	0.94
Acute abdominal pain (%)	206 (89.2)	246 (85.7)	0.24
Diarrhea (%)	48 (20.8)	30 (10.5)	0.001
Bloody stool (%) (macroscopic)	25 (10.8)	18 (6.3)	0.062
Vomiting (%)	30 (13.0)	18 (6.3)	0.009
Bowel emptying * (%)	68 (29.4)	47 (16.4)	<0.001
Shock (%)	43 (18.6)	34 (11.8)	0.031

AMI; acute mesenteric ischemia, IQR; interquartile range. * diarrhea and/or vomiting.

**Table 3:** Comparison of laboratory data at admission between patients with arterial occlusive AMI and non-AMI groups.

	Arterial Occlusive AMI (*n* = 231)	Non-AMI (*n* = 287)	*p*-Value
White blood cell count, ×10^9^/L (median, IQR)	16.2 (11.4–20.5) (*n* = 225)	12.5 (8.0–16.8) (*n* = 277)	<0.001
CRP, mg/L (median, IQR)	95 (21–216) (*n* = 188)	44 (7.5–118.5) (*n* = 201)	<0.001
eGFR, ml/min/1.73 m^2^ (median, IQR)	50 (26–80) (*n* = 146)	60 (36–85) (*n* = 206)	0.083
ASAT, U/L (median, IQR)	39 (23–73) (*n* = 188)	28 (19–55) (*n* = 187)	0.001
Amylase, U/L (median, IQR)	66 (41–152) (*n* = 107)	57 (34–122) (*n* = 140)	0.16
Troponin T, ng/L (median, IQR)	31 (13–125) (*n* = 106)	38 (10–183) (*n* = 64)	0.90
pH (median, IQR)	7.33 (7.23–7.41) (*n* = 187)	7.37 (7.27–7.42) (*n* = 209)	0.12
Base excess, (median, IQR)	−5.0 (−11.2–0.0) (*n* = 138)	−1.8 (−8.0–1.2) (*n* = 203)	0.014
D-dimer, mg/L (median, IQR)	4.0 (1.2–9.0) (*n* = 71)	4.5 (1.3–8.2) (*n* = 34)	0.73
Time point 1 *. Lactate, mmol/L (median, IQR)	1.6 (1.1–2.7) (*n* = 71)	1.7 (1.3–3.0) (*n* = 37)	0.40
Time point 2 **. Lactate, mmol/L (median, IQR)	1.7 (1.2–2.8) (*n* = 80)	2.2 (1.2–3.2) (*n* = 42)	0.54
Time point 3 ***. Lactate, mmol/L (median, IQR)	2.2 (1.3–4.0) (*n* = 98)	2.3 (1.4–3.7) (*n* = 75)	0.52
Time point 4 ****. Lactate, mmol/L (median, IQR)	3.2 (1.7–6.9) (*n* = 196)	2.4 (1.5–4.8) (*n* = 212)	0.034
Change in Lactate, mmol/L (median, IQR) between time points 4 and 1	0.0 (0.0–0.87) (*n* = 70)	0.50 (−0.22–2.15) (*n* = 34)	0.27
Change in Lactate, mmol (median, IQR) between time points 4 and 3	0.0 (0.0–1.20) (*n* = 96)	0.30 (−0.05–1.60) (*n* = 73)	0.43

AMI; acute mesenteric ischemia, IQR; interquartile range, CRP; c-reactive protein, eGFR; estimated glomerular filtration rate, ASAT; aspartate aminotransferase. * (48–72 h before diagnosis). ** (24–48 h before diagnosis). *** (12–24 h before diagnosis). **** (0–12 h before diagnosis).

**Table 4:** Variables associated with arterial occlusive AMI compared to non-AMI group in multivariable analysis.

Variable	Multivariable Logistic Regression	
	OR (95% CI)	*p*-Value
Current smoking	2.56 (1.31–5.03)	0.006
Hypertension	2.08 (1.09–3.97)	0.027
Atherosclerotic disease	0.70 (0.39–1.27)	0.24
Atrial fibrillation	1.58 (0.84–2.99)	0.16
White blood cell count, ×10^9^/L	1.54 * (1.14–2.08)	0.005
CRP, mg/L	1.19 * (0.91–1.56)	0.21
ASAT, U/L	1.34 * (0.94–1.91)	0.10
Bowel emptying **	3.25 (1.59–6.63)	0.001

AMI; acute mesenteric ischemia, OR; odds ratio, CI; confidence interval, CRP; c-reactive protein, ASAT; aspartate aminotransferase. * OR was expressed per one standard deviation increment. ** diarrhea and/or vomiting.

## Data Availability

The data that support the findings of this study are available on request from the corresponding author (S.A).

## Supplementary Materials

The following supporting information can be downloaded at https://www.mdpi.com/article/10.3390/diagnostics14232705/s1, Table S1. Univariate logistic regression towards the outcome, arterial occlusive AMI, for 21 pre-specified candidate predictors. Table S2. Trade-off from using the model based on a range of treatment thresholds for the prediction model-derived probability of arterial occlusive AMI. Figure S1. Calibration plot for the full arterial occlusive acute mesenteric ischemia (AMI) occlusion prediction model based on internal validation using 500 sample bootstrapping and the five model variables: Serum white cell blood count (WBC), bowel emptying history, serum C-reactive protein (CRP), arterial hypertension and current smoker. Figure S2. Nomogram for calculating the individual probability for arterial occlusive acute mesenteric ischemia (AMI) among adult patients presenting with abdominal pain in the emergency department. Summarize the scores for each of the five variables to a total score for a corresponding probability assessment [35,36].

## Appendix A

**Table A1:** Comparison of clinical background data between patients with arterial occlusive AMI and SBO.

	Arterial Occlusive AMI (*n* = 231)	SBO (*n* = 128)	*p*-Value
Age, years (median, IQR)	71 (60–80)	70 (58–80)	0.47
Female gender (%)	100 (43.3)	67/125 (53.6)	0.063
Body Mass Index, kg/m^2^ (median, IQR)	24.4 (21.3–27.7) (*n* = 180)	24.2 (20.3–28.1) (*n* = 101)	0.78
Disability (%)	49/219 (22.4)	32/121 (26.4)	0.040
Current smoking (%)	70/180 (38.9)	20/99 (20.2)	0.001
Atherosclerotic disease (%)	100/217 (46.1)	35/122 (28.7)	0.002
Hypertension (%)	166/224 (74.1)	70/125 (56.0)	<0.001
Myocardial infarction (%)	48/221 (21.7)	10/124 (8.1)	0.001
Previous thromboembolic arterial event (%)	25/214 (11.7)	3/124 (2.4)	0.003
Charlson comorbidity index (median, IQR)	4 (3–6) (*n* = 212)	4 (2–5) (*n* = 121)	0.001
Anticoagulant drugs (%)	70/217 (32.3)	25/127 (19.7)	0.012
Antiplatelet drugs (%)	77/212 (36.3)	30/126 (23.8)	0.017
Statins (%)	76/213 (35.7)	36/126 (28.6)	0.18

AMI; acute mesenteric ischemia, SBO; strangulating bowel obstruction, IQR; interquartile range.

**Table A2:** Comparison of clinical presentation between patients with arterial occlusive AMI and SBO.

	Arterial Occlusive AMI (*n* = 231)	SBO (*n* = 128)	*p*-Value
Pre-hospital symptom duration, hours (median; IQR)	24 (9–48) (*n* = 172)	24 (8–48) (*n* = 87)	0.93
New atrial fibrillation (%)	23 (10.0)	4 (3.1)	0.019
Atrial fibrillation (%)	84 (36.4)	28 (21.9)	0.005
Acute abdominal pain (%)	206 (89.2)	120 (93.8)	0.15
Diarrhea (%)	48 (20.8)	7 (5.5)	<0.001
Bloody stool (%) (macroscopic)	25 (10.8)	5 (3.9)	0.023
Vomiting (%)	30 (13.0)	13 (10.2)	0.43
Bowel emptying * (%)	68 (29.4)	20 (15.6)	0.004
Shock (%)	43 (18.6)	5 (3.9)	<0.001

AMI; acute mesenteric ischemia, SBO; strangulating bowel obstruction, IQR; interquartile range. * diarrhea and/or vomiting.

**Table A3:** Comparison of laboratory data between patients with arterial occlusive AMI and SBO.

	Arterial Occlusive AMI (*n* = 231)	SBO (*n* = 128)	*p*-Value
White blood cell count, ×10^9^/L (median, IQR)	16.2 (11.4–20.5) (*n* = 225)	12.4 (8.2–16.0) (*n* = 123)	<0.001
CRP, mg/L (median, IQR)	95 (21–216) (*n* = 188)	36 (6–112) (*n* = 83)	<0.001
eGFR, ml/min/1.73 m^2^ (median, IQR)	50 (26–80) (*n* = 146)	60 (39–86) (*n* = 85)	0.044
ASAT, U/L (median, IQR)	39 (23–73) (*n* = 188)	24 (18–32) (*n* = 78)	<0.001
Amylase, U/L (median, IQR)	66 (41–152) (*n* = 107)	67 (37–123) (*n* = 55)	0.63
Troponin T, ng/L (median, IQR)	31 (13–125) (*n* = 106)	30 (10–105) (*n* = 29)	0.54
pH (median, IQR)	7.33 (7.23–7.41) (*n* = 187)	7.38 (7.34–7.42) (*n* = 101)	<0.001
Base excess, (median, IQR)	−5.0 (−11.0–0.0) (*n* = 137)	−0.80 (−5.50–2.0) (*n* = 99)	<0.001
D-dimer, mg/L (median, IQR)	4.0 (1.3–10.0) (*n* = 71)	1.4 (0.3–5.0) (*n* = 15)	0.009
Time point 1 *. Lactate, mmol/L (median, IQR)	1.6 (1.1–2.7) (*n* = 71)	1.6 (0.6–1.8) (*n* = 11)	0.40
Time point 2 **. Lactate, mmol/L (median, IQR)	1.7 (1.2–2.8) (*n* = 80)	1.4 (0.6–2.5) (*n* = 11)	0.33
Time point 3 ***. Lactate, mmol/L (median, IQR)	2.2 (1.3–4.0) (*n* = 98)	2.0 (1.4–2.7) (*n* = 32)	0.26
Time point 4 ****. Lactate, mmol/L (median, IQR)	3.2 (1.7–6.9) (*n* = 196)	2.2 (1.5–3.5) (*n* = 98)	<0.001

AMI; acute mesenteric ischemia, SBO; strangulating bowel obstruction, CRP; c-reactive protein, eGFR; estimated glomerular filtration rate, ASAT; aspartate aminotransferase. * (48–72 h before diagnosis). ** (24–48 h before diagnosis). *** (12–24 h before diagnosis). **** (0–12 h before diagnosis).

**Table A4:** Comparison of clinical background data between patients with embolic arterial occlusive AMI and non-AMI.

	Embolic Arterial Occlusive AMI (*n* = 61)	Non-AMI (*n* = 287)	*p*-Value
Age, years (median, IQR)	76 (66–83)	69 (56–59)	0.008
Female gender (%)	33 (43.1)	138/284 (48.6)	0.45
Body Mass Index, kg/m^2^ (median, IQR)	25.2 (23.3–28.4) (*n* = 49)	24.7 (22.0–27.7) (*n* = 216)	0.15
Disability (%)	15 (24.6)	75/269 (27.9)	0.62
Current smoking (%)	10/55 (16.4)	51/225 (22.7)	0.47
Atherosclerotic disease (%)	25 (41.0)	96/269 (35.7)	0.44
Hypertension (%)	49 (80.3)	169/280 (60.4)	0.003
Myocardial infarction (%)	12 (19.7)	33/274 (12.0)	0.11
Previous thromboembolic arterial event (%)	5/59 (8.5)	12/272 (4.4)	0.20
Charlson comorbidity index (median, IQR)	4 (3–6) (*n* = 58)	4 (2–5) (*n* = 268)	0.083
Anticoagulant drugs (%)	20/60 (33.3)	56/270 (20.7)	0.036
Antiplatelet drugs (%)	16/60 (26.7)	76/271 (28.0)	0.83
Statins (%)	22/60 (36.7)	85/270 (31.5)	0.44

AMI; acute mesenteric ischemia, IQR; interquartile range.

**Table A5:** Comparison of clinical presentation at admission between patients with embolic arterial occlusive AMI and non-AMI.

	Embolic Arterial Occlusive AMI (*n* = 61)	Non-AMI (*n* = 287)	*p*-Value
Pre-hospital symptom duration, hours (median; IQR)	18 (4–48) (*n* = 47)	24 (6–48) (*n* = 190)	0.19
New atrial fibrillation (%)	11 (18.0)	10 (3.5)	<0.001
Atrial fibrillation (total) (%)	41 (67.2)	65 (22.6)	<0.001
Acute abdominal pain (%)	55 (90.2)	246 (85.7)	0.36
Diarrhea (%)	15 (24.6)	30 (10.5)	0.003
Bloody stool (%)	10 (16.4)	18 (6.3)	0.008
Vomiting (%)	10 (16.4)	18 (6.3)	0.008
Bowel emptying * (%)	19 (31.1)	47 (16.4)	0.008
Shock (%)	14 (23.0)	34 (11.8)	0.022

AMI; acute mesenteric ischemia, IQR; interquartile range. * diarrhea and/or vomiting.

**Table A6:** Comparison of laboratory data between patients with embolic arterial occlusive AMI and non-AMI.

	Embolic Arterial Occlusive AMI (*n* = 61)	Non-AMI (*n* = 287)	*p*-Value
White blood cell count, ×10^9^/L (median, IQR)	16.6 (11.4–19.0) (*n* = 59)	12.5 (8.0–16.8) (*n* = 277)	<0.001
CRP, mg/L (median, IQR)	73 (19.2–174.8) (*n* = 52)	44 (7.5–118.5) (*n* = 201)	0.026
eGFR, ml/min/1.73 m^2^ (median, IQR)	56 (30–82) (*n* = 40)	60 (36–85) (*n* = 206)	0.41
ASAT, U/L (median, IQR)	34 (25–61) (*n* = 54)	28 (19–55) (*n* = 187)	0.12
Amylase, U/L (median, IQR)	69 (50–138) (*n* = 30)	57 (34–122) (*n* = 140)	0.15
Troponin T, ng/L (median, IQR)	39 (13–188) (*n* = 31)	38 (10–183) (*n* = 64)	0.82
pH (median, IQR)	7.34 (7.26–7.42) (*n* = 52)	7.37 (7.27–7.42) (*n* = 209)	0.92
Base excess, (median, IQR)	−2.9 (−8.0–0.0) (*n* = 39)	−1.8 (−8.0–1.2) (*n* = 203)	0.40
D-dimer, mg/L (median, IQR)	5.0 (2.0–9.5) (*n* = 21)	4.5 (1.3–8.2) (*n* = 34)	0.23
Time point 1 *. Lactate, mmol/L (median, IQR)	1.7 (1.3–2.3) (*n* = 15)	1.7 (1.3–3.0) (*n* = 37)	0.68
Time point 2 **. Lactate, mmol/L (median, IQR)	1.8 (1.3–2.9) (*n* = 19)	2.2 (1.2–3.2) (*n* = 42)	0.95
Time point 3 ***. Lactate, mmol/L (median, IQR)	2.4 (1.7–3.1) (*n* = 23)	2.3 (1.4–3.7) (*n* = 75)	0.98
Time point 4 ****. Lactate, mmol/L (median, IQR)	3.0 (1.8–5.4) (*n* = 54)	2.4 (1.5–4.8) (*n* = 212)	0.30
Change in Lactate, mmol/L (median, IQR) between time points 4 and 1	0.2 (−0.1–0.8) (*n* = 15)	0.50 (−0.22–2.15) (*n* = 34)	0.28

AMI; acute mesenteric ischemia, IQR; interquartile range, CRP; c-reactive protein, ASAT; aspartate aminotransferase. * (48–72 h before diagnosis). ** (24–48 h before diagnosis). *** (12–24 h before diagnosis). **** (0–12 h before diagnosis).
