# Supplementary material for: Diagnostic Performance of Clinical and Routine Laboratory Data in Acute Mesenteric Arterial Occlusion—An International Multicenter Study
Source: Diagnostics (Basel). 2024 Nov 30;14(23):2705. doi: 10.3390/diagnostics14232705 (PMC11640103; doi:10.3390/diagnostics14232705)
Supplement: Supplementary file 1 [file diagnostics-14-02705-s001.zip › diagnostics-3254750-supplementary.pdf]

Supplementary Materials - Prediction model development

Aim

We aimed at developing a prediction model based on clinical and laboratory parameters from the AMESI study to answer the clinical question "Should this patient with acute abdominal pain and suspected arterial occlusive acute mesenteric ischemia (AMI) go through an immediate diagnostic computed tomography (CT) angiography or could a CT scan be safely spared in this patient?".

Statistical analysis

We pre-specified primary, secondary and exploratory candidate variables to be included in the prediction model based on clinical experience and former known association with the outcome (i.e. AMI). We then used a trade-off between clinical feasibility and apparent predictive performance in the dataset at hand to choose the variables to be included in the final model. Candidate variables were analyzed univariately towards the outcome using logistic regression on their original scale. We limited the variables in the final model to five to be clinically manageable and to avoid overfitting. We considered non-linearity among predictors. We investigated missingness and type of missingness and considered multiple imputation if exceeding 5% in any one variable. Influential observations were winsorised to a clinically reasonable range. All statistical analyses were performed using Stata (Stata/MP 18.0, StataCorp, Texas, USA). The prediction model development and validation are based on the work by Steyerberg *et al.* [35, 36]

Results

We pre-specified 21 candidate predictors whose univariate association with the outcome can be seen in Table S1. Serum white cell blood count, bowel emptying history, serum C-reactive protein arterial hypertension and current smoker were the five parameters with the strongest association towards the outcome (pseudo R^2 range 0.015 to 0.030) and were all considered clinically feasible. We found no non-linear relationships among the included variables. There were considerable missingness in the included variables (range 0 to 25%). We considered these variables to be missing at random and we performed multiple imputation using correlated non-missing variables and 20 iteration chained equations.

Table S1. Univariate logistic regression towards the outcome, arterial occlusive AMI, for 21 pre-specified candidate predictors.

Variable	Pseudo R^2	Missing
Primary		
Serum white blood cell count (continuous)	0.0299	3%
Previous arterial thrombosis (yes/no)	0.0148	6%
Previous atrial fibrillation (yes/no)	0.0118	4%
Previous atherosclerosis (yes/no)	0.0081	6%
Age (continuous)	0.0074	0%
Acute abdominal pain (yes/no)	0.0020	0%
Secondary		
Bowel emptying history, stool or vomit (yes/no)	0.0177	0%

Previous myocardial infarction (yes/no)	0.0122	4%
Exploratory		
Serum C-reactive protein (continuous)	0.0268	25%
Arterial hypertension (yes/no)	0.0154	3%
Previous anti-coagulant therapy (yes/no)	0.0124	6%
APACHE II score (continuous)	0.0087	44%
Serum Creatinine (continuous)	0.0069	6%
SOFA score (continuous)	0.0060	41%
Previous anti-platelet therapy (yes/no)	0.0057	7%
Symptom duration (continuous)	0.0057	30%
pH (continuous)	0.0038	24%
Biological sex (yes/no)	0.0020	1%
Body mass index (continuous)	0.0006	24%
Current smoker (yes/no)	0.0300	22%
Tropinin T (continuous)	0.0003	67%

AMI; acute mesenteric ischemia, SOFA; Sequential organ failure assessment, APACHE; Acute physiology and chronic health evaluation.

We evaluated the performance of the final prediction model using internal validation by 500 sample bootstrapping. The model had a raw fit of pseudo R^2 0.146 and an optimism-adjusted C-statistic (i.e., area under the receiver operating characteristics curve, AUROC) of 0.718. The model was well-calibrated based on expected:observed ratio, slope and calibration in the large (Figure S1).

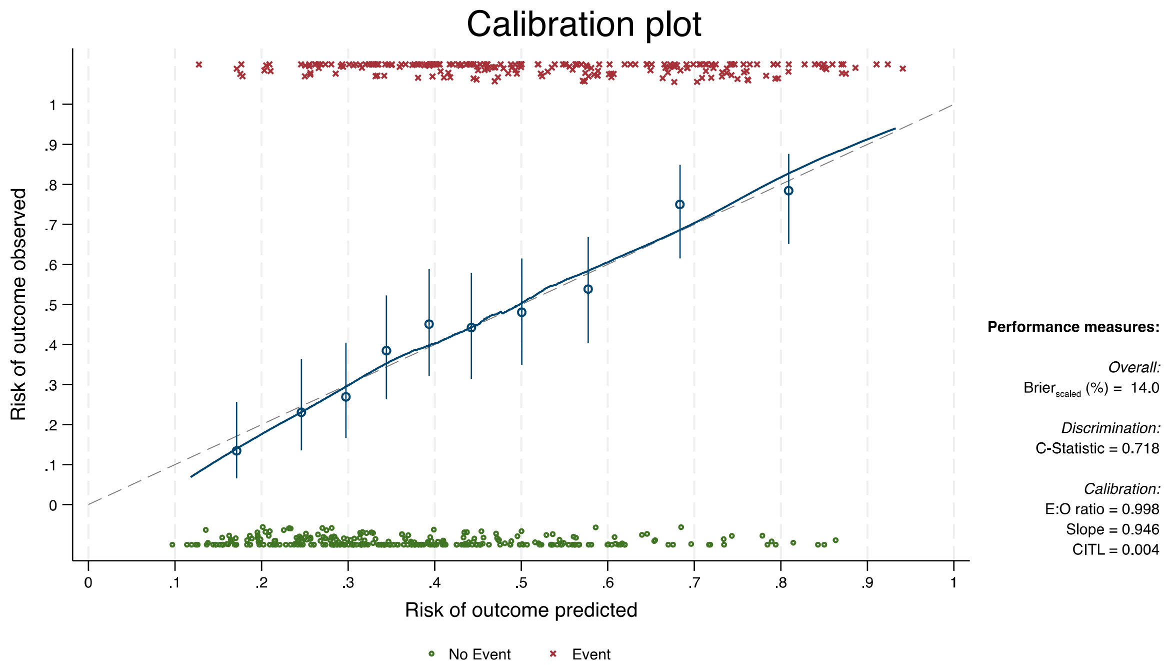

Figure S1. Calibration plot for the full arterial occlusive acute mesenteric ischemia (AMI) occlusion prediction model based on internal validation using 500 sample bootstrapping and the five model variables: Serum white

cell blood count (WBC), bowel emptying history, serum C-reactive protein (CRP), arterial hypertension and current smoker.

The individual probability for arterial occlusive AMI can be straightforwardly calculated using the nomogram (Figure S2).

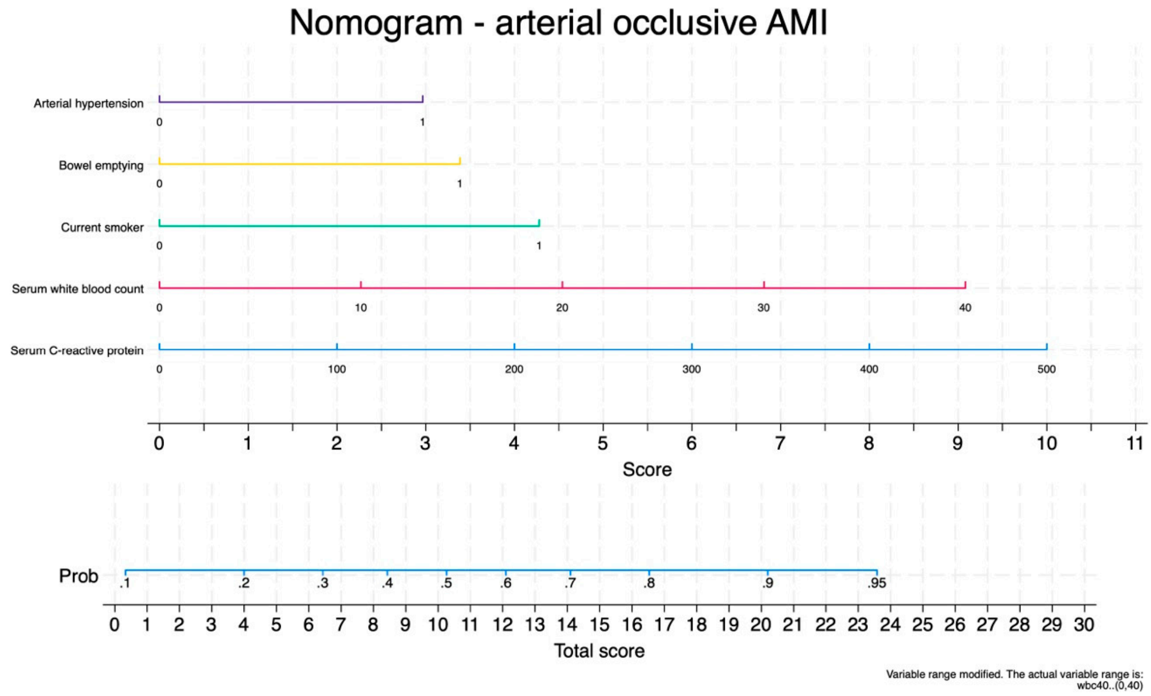

Figure S2. Nomogram for calculating the individual probability for arterial occlusive acute mesenteric ischemia (AMI) among adult patients presenting with abdominal pain in the emergency department. Summarize the scores for each of the five variables to a total score for a corresponding probability assessment.

All patients in the cohort had a model-derived probability for arterial occlusive AMI above 10%. Probabilities ranged from 10.8% to 86.5% in patients with a negative outcome and from 13.8% to 94.2% in patients with a positive outcome, with significant overlap (Figure S1). The trade-off from a theoretical implementation of the prediction model across different treatment thresholds (i.e., across a range of model-derived probabilities for arterial occlusive AMI where a CT scan is recommended to be performed) can be seen in Table S2. There is a theoretical, but highly data-driven, potential to save 11 CT scans without any missed cases at a treatment threshold of 13.7%. Above that threshold, there was a diminishing return regarding the number of CT scans saved per missed case for each incremental increase in treatment threshold.

Table S2. Trade-off from using the model based on a range of treatment thresholds for the prediction model-derived probability of arterial occlusive AMI

Treatment threshold	False negative	True negative	Number of saved CT scans per missed case
10%	0	0	0
13.7%	0	11	
15%	2	16	8.0
20%	6	43	7.2
25%	16	71	4.4
30%	30	108	3.6
40%	65	178	2.7
50%	113	223	2.0

AMI; acute mesenteric ischemia, CT; computed tomography

Conclusion

Based on the high mortality of arterial occlusive AMI and the low level of harm from a CT scan, it is our interpretation that the herein developed prediction model cannot be recommended to be used to avoid CT scanning at any treatment threshold in patients at the ED with acute abdominal pain and suspected arterial occlusive AMI.

References

(35) Steyerberg, E. W. Clinical Prediction Models. *Statistics for Biology and Health* **2009**. DOI: 10.1007/978-0-387-77244-8.

(36) Steyerberg, E. W.; Vergouwe, Y. Towards better clinical prediction models: seven steps for development and an ABCD for validation. *Eur Heart J* **2014**, *35* (29), 1925-1931. DOI: 10.1093/eurheartj/ehu207 From NLM Medline.
